# Onboard LiDAR–Camera Deployment Optimization for Pavement Marking Distress Fusion Detection

**DOI:** 10.3390/s25133875

**Published:** 2025-06-21

**Authors:** Ciyun Lin, Wenjian Sun, Ganghao Sun, Bown Gong, Hongchao Liu

**Affiliations:** 1 Department of Traffic Information and Control Engineering, Jilin University, Changchun 130022, China; linciyun@jlu.edu.cn (C.L.); sunwj22@mails.jlu.edu.cn (W.S.); sungh23@mails.jlu.edu.cn (G.S.); 2 Jilin Engineering Research Center for Intelligent Transportation System, Changchun 130022, China; 3 Department of Civil, Environmental and Construction Engineering, Texas Tech University, Lubbock, TX 79409, USA; hongchao.liu@ttu.edu

**Keywords:** sensor deployment optimization, pavement marking distress detection, multi-sensor data fusion, red fox optimization

## Abstract

Pavement markings, as a crucial component of traffic guidance and safety facilities, are subject to degradation and abrasion after a period of service. To ensure traffic safety, retroreflectivity and diffuse illumination should be above the minimum thresholds and required to undergo inspection periodically. Therefore, an onboard light detection and ranging (LiDAR) and camera deployment optimization method is proposed for pavement marking distress detection to adapt to complex traffic conditions, such as shadows and changing light. First, LiDAR and camera sensors’ detection capability was assessed based on the sensors’ built-in features. Then, the LiDAR–camera deployment problem was mathematically formulated for pavement marking distress fusion detection. Finally, an improved red fox optimization (RFO) algorithm was developed to solve the deployment optimization problem by incorporating a multi-dimensional trap mechanism and an improved prey position update strategy. The experimental results illustrate that the proposed method achieves 5217 LiDAR points, which fall on a 0.58 m pavement marking per data frame for distress fusion detection, with a relative error of less than 7% between the mathematical calculation and the field test measurements. This empirical accuracy underscores the proposed method’s robustness in real-world scenarios, effectively mitigating the challenges posed by environmental interference.

## 1. Introduction

Pavement markings play an essential role in enhancing traffic safety and providing traffic information for guidance, and they regulate driver behavior [1,2]. Furthermore, pavement markings provide a foundation criterion in advanced driver assistance system (ADAS) development, such as lane-keeping, fatigue driving detection, and adaptive cruise control [3,4].

However, pavement markings may undergo distress, such as cracks, wear, and aging due to environmental erosion and vehicle loading [5,6]. To check whether pavement markings meet the minimum retroreflectivity requirements, traffic engineers usually use handheld laser reflectometers for on-site inspections. However, this human-controlled method often depends on engineers’ experience and suffers from low efficiency and high risk in field testing [7,8].

In existing research, camera sensors are used in pavement marking detection due to their high resolution and low cost, enabling the precise extraction of marking features through image processing techniques. However, they are sensitive to variations in ambient lighting, making them susceptible to changes in environmental illumination [9]. On the other hand, the light detection and ranging (LiDAR) sensor demonstrates strong robustness to external lighting changes and provides broader scanning coverage, but its sparse point cloud limits its effectiveness in fine feature detection applications, such as pavement marking distress detection [10,11,12]. To improve the coverage and point cloud density of LiDAR in pavement marking detection, onboard-mounted LiDAR parameters were optimized by constructing a deployment optimization model in our early work [11], and a marking distress detection method [6] was proposed based on high-quality point clouds. However, the method has a limited effect on the detection of distress signs with tiny dimensions (fine cracks, minor spalling, etc.). Given the advantages of vision cameras in capturing details and distinguishing small targets, this study further introduces cameras into the deployment optimization system to determine the optimal deployment strategy for multi-sensor fusion [13,14].

Synergistically optimizing the relative positions and viewing angles of LiDAR and cameras can help them overcome the limitations of their respective sensors to leverage their complementary strengths in the detection of large-scale marking lesions and small cracks in complex road environments. This is considered a cost-effective way to achieve robustness and accuracy. However, determining how to jointly optimize the deployment of heterogeneous sensors under a multi-sensor fusion framework to maximize the detection performance of pavement marking lesions is still a key challenge.

Therefore, an onboard LiDAR–camera deployment optimization method was proposed for pavement marking distress fusion detection. First, the onboard LiDAR and camera sensors’ detection range on pavement was evaluated based on the sensors’ built-in characteristics. Then, the LiDAR–camera deployment problem was mathematically formulated to achieve the largest overlap area of detection and most points fall on pavement marking for fusion detection. Finally, an improved red fox optimization (RFO) algorithm was developed for LiDAR–camera mounting parameter optimization by leveraging a multi-dimensional trap mechanism to bound the search space and enforce physical deployment constraints, and an improved prey position update strategy was created to ensure monotonic convergence toward globally optimal configurations.

The main contributions of this paper can be summarized as follows:

(1) An onboard LiDAR–camera co-deployment mathematical model was proposed for pavement marking distress fusion detection to maximize the fusion detection region and point cloud density.

(2) An improved red fox optimization (RFO) algorithm was developed to adapt the constraints in onboard sensor deployment and speed up the convergence rate of the problem solution.

(3) The experimental results show that the proposed method achieves a relative error below 7% between mathematical evaluation and field measurements, and they illustrate the robustness of pavement marking distress detection in environments with shadows and changing light.

The rest of this paper is organized as follows: Section 2 reviews the related research on pavement marking detection and sensor deployment optimization. Section 3 presents the onboard LiDAR–camera deployment optimization problem and solution methodology. Section 4 presents the experiments that were conducted to illustrate the performance of the proposed method. Section 5 concludes this study with a summary and possible future work.

## 2. Related Works

To draw a clear distinction between this study and previous research, the literature on pavement marking detection and sensor deployment optimization is reviewed in this section.

### 2.1. Pavement Marking Detection

The existing research on pavement marking detection can be divided into studies focusing on image-based methods and LiDAR-based methods according to the sensor used for data collection [15,16].

Image-based methods can be further categorized into feature-based [17,18], model-based [3,4], and learning-based [19,20] methods. For instance, Borkar [17] converted an image into a grayscale map and used marker edges as features through canny edge extraction for pavement marking detection. Zhou [21] established a geometric detection model for pavement marking detection based on local Hough transform and model matching. Hani [20] proposed a deep learning framework based on Fast-RCNN for pavement marking identification. However, these methods focus on pavement marking outline detection, which cannot be used in pavement marking distress detection. In addition, the accuracy and robustness of image-based methods are always affected by the light conditions and shadows.

In the current study, the LiDAR sensor was also used for pavement marking distress detection. Three-dimensional (3D) point cloud data extracted from the LiDAR sensor was projected into a two-dimensional (2D) plane to form an image. Then, pavement marking distress was detected by leveraging image-based algorithms [6,22]. For instance, Li [12] utilized a laser scanner to obtain high-resolution laser images, identifying lane markings’ contours through closing operations and a marching square algorithm. They ultimately employed a linear support vector machine method for geometric feature extraction and the reconstruction of pavement markings. Zhang [22] mapped the three-dimensional data of the identified areas onto a two-dimensional image and finally accurately extracted the crosswalk marking regions using a convolutional neural network.

Some researchers use a point cloud to reconstruct a 3D pavement marking map and recognize the distress based on the point cloud features [23]. For instance, Rastiveis [24] identified pavement marking areas using the Hough transform and fuzzy inference methods based on laser intensity information and point cloud positions. Cheng [23] used the intensity information of laser point clouds as features and constructed an unsupervised learning network, U-Net, to detect pavement markings.

However, sparse point clouds make it difficult to detect various types and sizes of pavement marking distress due to their low resolution.

### 2.2. Sensor Deployment Optimization

The rich visual information provided by the camera can help address the limitations of LiDAR in surface texture perception, while the LiDAR sensor can compensate for the limitations of images with shadows. LiDAR–camera data fusion can achieve more comprehensive and accurate pavement marking distress detection. However, the research on LiDAR–camera fusion for pavement marking distress detection is limited.

To leverage the sensor’s performance to the maximum extent, its deployment position and pose should be optimized according to the objective application. In existing research, the deployment of homogeneous sensors in a network was optimized to obtain the best location and pose to expand the coverage, improve accuracy, and reduce the investment cost. Hörster [25] proposed a linear programming-based approach to optimize multi-camera deployments to maximize the detection range by considering the sensor number, location, and cost. Geissler [26] used genetic algorithms to maximize camera sensors’ FOV in dynamic occlusion scenarios. Du [27] proposed a sensor deployment method for vehicle detection and tracking under different traffic densities and vehicle compositions, which can reduce the sensor’s investment cost and expand detection coverage.

Heterogeneous multi-sensor deployment optimization always focuses on the strength of the sensing ability. Dey [28] proposed a VESPA framework to optimize the deployment position and orientation of onboard cameras and radars to extend the coverage and enhance detection accuracy and reliability. Cao [29] proposed a method for the optimal deployment of cameras and radars on road networks using the bisection method and the AO–GI–MO algorithm, which effectively improved traffic accident detection. Li [30] proposed an optimal deployment method for roadside LiDAR sensors and cameras by comparing experimental data from multiple application scenarios and performing object detection and recognition using YOLOv5s and PointPillars, significantly improving the accuracy of blind spot detection and vehicle occlusion issues.

However, to the best of our knowledge, the problems of the onboard LiDAR–camera deployment optimization for pavement marking distress detection have not been addressed.

## 3. Methodology

### 3.1. Problem Formulation

**Figure 1:**
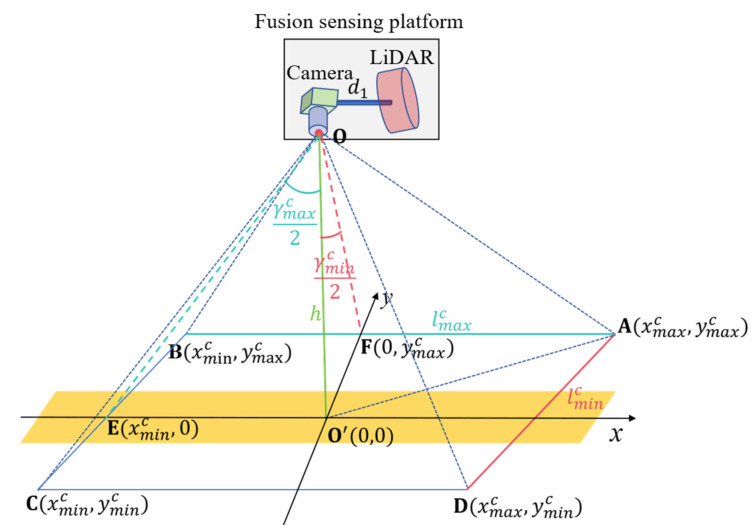
The schematic diagram of the camera’s scanning range.

Assuming that the projected coordinates origin on the ground is $O^{’}\left(0,0\right)$, the camera’s scanning range of the road pavement can be defined as $S_{AR}^{C}=\left\{A,B,C,D\right\}$, as shown in Figure 1.

According to the camera sensor’s built-in field of view (FOV), denoted as $\left(\gamma_{min}^{c},\gamma_{max}^{c}\right)$, and its deployment height h, the camera’s scanning range can be rewritten as

(1) $$S_{AR}^{C}=\left\{\begin{array}{c} \left(x_{max}^{c},y_{max}^{c}\right)\\ \left(x_{min}^{c},y_{max}^{c}\right)\\ \left(x_{min}^{c},y_{min}^{c}\right)\\ \left(x_{max}^{c},y_{min}^{c}\right) \end{array}\right.=\left\{\begin{array}{c} A\left(h\cdot \tan\left(\frac{\gamma_{min}^{c}}{2}\right),h\cdot \tan\left(\frac{\gamma_{min}^{c}}{2}\right)\right)\\ B\left(-h\cdot \tan\left(\frac{\gamma_{max}^{c}}{2}\right),h\cdot \tan\left(\frac{\gamma_{min}^{c}}{2}\right)\right)\\ C\left(-h\cdot \tan\left(\frac{\gamma_{max}^{c}}{2}\right),-h\cdot \tan\left(\frac{\gamma_{min}^{c}}{2}\right)\right)\\ D\left(h\cdot \tan\left(\frac{\gamma_{min}^{c}}{2}\right),-h\cdot \tan\left(\frac{\gamma_{min}^{c}}{2}\right)\right) \end{array}\right.$$

where $x_{min}^{c},x_{max}^{c}$ and $y_{min}^{c},y_{max}^{c}$ are the upper and lower coordinates of the camera’s detection range in the X and Y axes, respectively.

The pavement marking detection length L of the camera can be mathematically formulated as

(2) $$L=\left[2h\cdot tan\left(\frac{\gamma_{min}^{c}}{2}\right),2h\cdot \tan\left(\frac{\gamma_{max}^{c}}{2}\right)\right]$$

**Figure 2:**
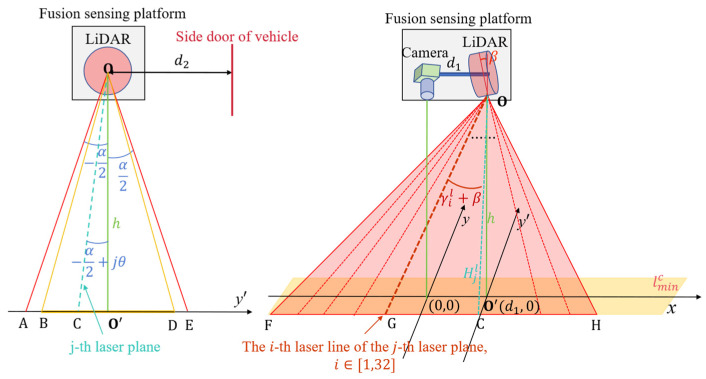
The schematic diagram of the LiDAR sensor’s scanning range.

The distance between the LiDAR sensor and the camera is $d_{1}$, and the coordinates’ origin of the LiDAR sensor on the ground can be presented as $O^{’}\left(d_{1},0\right)$, as shown in Figure 2.

According to the LiDAR sensor’s horizontal FOV $\left(-\frac{\alpha }{2},\frac{\alpha }{2}\right)$, resolution θ, and deployment height h, the LiDAR sensor’s scanning range can be expressed as

(3) $$\left\{\begin{array}{c} x_{i,j}^{l}=\frac{h\cdot tan\left(\gamma_{i}^{l}+\beta \right)}{\cos\left(-\frac{\alpha }{2}+j\theta \right)}+d_{1}\\ y_{i,j}^{l}=h\cdot \tan\left(-\frac{\alpha }{2}+j\theta \right) \end{array}\right.$$

where i is the ID of the LiDAR laser beam. j is the point within the scanning resolution sequence. $\left(x_{i,j}^{l},y_{i,j}^{l}\right)$ are the coordinates of the laser point.

The angular point of the LiDAR sensor’s scanning range $S_{AR}^{L}$ can be expressed as

(4) $$S_{AR}^{L}=\left\{\begin{array}{c} \left(x_{max}^{l},y_{max}^{l}\right)\\ \left(x_{min}^{l},y_{max}^{l}\right)\\ \left(x_{min}^{l},y_{min}^{l}\right)\\ \left(x_{max}^{l},y_{min}^{l}\right) \end{array}\right.=\left\{\begin{array}{c} A^{’\left(\frac{h\cdot tan\left(\gamma_{min}^{l}+\beta \right)}{\cos\left(\frac{\alpha }{2}\right)}+d_{1},h\cdot tan\left(\frac{\alpha }{2}\right)\right)}\\ B^{’\left(\frac{h\cdot tan\left(\gamma_{min}^{l}+\beta \right)}{\cos\left(\frac{\alpha }{2}\right)}+d_{1},-h\cdot tan\left(\frac{\alpha }{2}\right)\right)}\\ C^{’\left(\frac{h\cdot tan\left(\gamma_{min}^{l}+\beta \right)}{\cos\left(\frac{\alpha }{2}\right)},h\cdot tan\left(\frac{\alpha }{2}\right)\right)}\\ D^{’}\left(\frac{h\cdot tan\left(\gamma_{min}^{l}+\beta \right)}{\cos\left(\frac{\alpha }{2}\right)}+d_{1},-h\cdot tan\left(\frac{\alpha }{2}\right)\right) \end{array}\right.$$

where $x_{min}^{l},x_{max}^{l}$ and $y_{min}^{l},y_{max}^{l}$ are the upper and lower coordinates of the LiDAR sensor’s detection range in the X and Y axes, respectively.

The overlap area $S_{OA}$ of the LiDAR–camera scanning range can be formulated as

(5) $$S_{OA}=S_{AR}^{C}\cap S_{AR}^{L}=\left\{\begin{array}{c} x_{min}^{F}=\max\left\{x_{min}^{l},x_{min}^{c}\right\}\\ x_{max}^{F}=\min\left\{x_{max}^{l},x_{max}^{c}\right\}\\ y_{min}^{F}=\max\left\{y_{min}^{l},y_{min}^{c}\right\}\\ y_{max}^{F}=\min\left\{y_{max}^{l},y_{max}^{c}\right\} \end{array}\right.$$

where $x_{min}^{F},x_{max}^{F}$ and $y_{min}^{F},y_{max}^{F}$ are the upper and lower coordinates of the LiDAR–camera sensor’s detection overlap area in the X and Y axes, respectively.

To improve the efficiency and accuracy of pavement marking distress detection and evaluation, the onboard LiDAR and camera sensor’s overlap scanning area for pavement marking should meet the following criteria:

1. The pavement marking coverage area for data fusion should be as large as possible. That is, the detection length for pavement marking L should be as long as possible due to the width of the pavement marking being fixed.

(6) $$\max f_{1}\left(h,\alpha ,\beta \right)=\min\left\{x_{max}^{l},x_{max}^{c}\right\}-max\left\{x_{min}^{l},x_{min}^{c}\right\}$$

2. There should be as many laser points that fall on pavement marking as possible.

(7) $$\max f_{2}\left(h,\alpha ,\beta \right)=\sum_{i=1}^{m}\sum_{j=0}^{j=\frac{\alpha }{\theta }}n_{i,j}$$

(8) $$n_{i,j}=\left\{\begin{array}{c} 0,\;x_{i,j}^{l}\notin \left[x_{min}^{F},x_{min}^{F}\right]\;or\;y_{i,j}^{l}\notin \left[y_{min}^{F},y_{min}^{F}\right]\\ 1,\;x_{i,j}^{l}\in \left[x_{min}^{F},x_{min}^{F}\right]\;and\;y_{i,j}^{l}\in \left[y_{min}^{F},y_{min}^{F}\right] \end{array}\right.$$

where $n_{i,j}$ indicates whether the laser point $\left(x_{i,j}^{l},y_{i,j}^{l}\right)$ fall within the overlap area.

**Figure 3:**
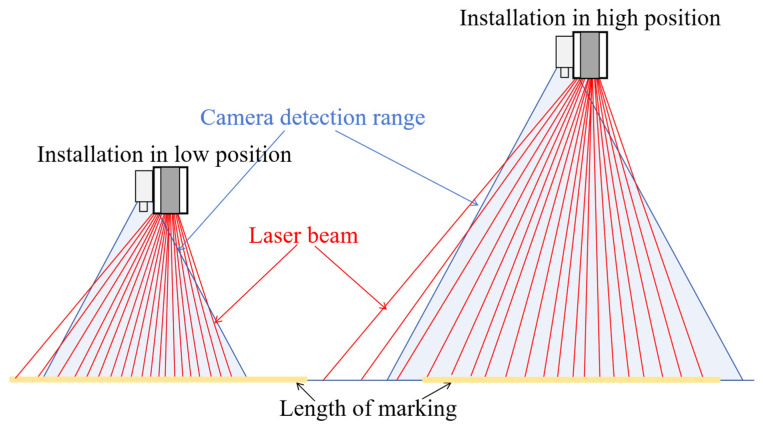
The schematic diagram of the inverse relationship between the sensor’s installation height and the pavement marking detection length.

According to the sensor’s mechanical structure and working principle, the pavement marking scanning length increases as the sensor’s installation height h increases. Conversely, the point cloud density and pixel resolution of the pavement marking decreases as the sensor’s installation height h increases, as shown in Figure 3.

**Figure 4:**
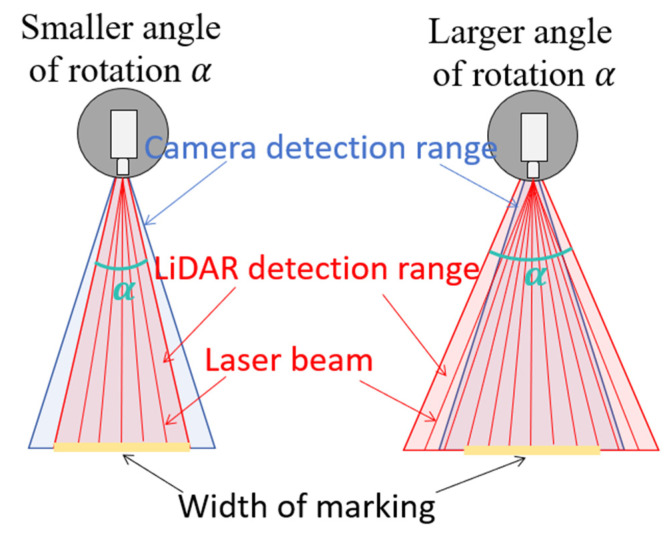
The schematic diagram of the inverse relationship between the LiDAR sensor’s rotation angle α and the pavement scanning width.

The LiDAR sensor’s rotation angle α directly determines the scanning width of the pavement when the LiDAR sensor is installed vertically. A smaller α value results in a narrower scanning width, which may lead to incomplete coverage of the markings. On the other hand, an increase in α broadens the scanning width, leading to sparser point clouds in pavement marking and more invalid point clouds in data processing, as shown in Figure 4.

**Figure 5:**
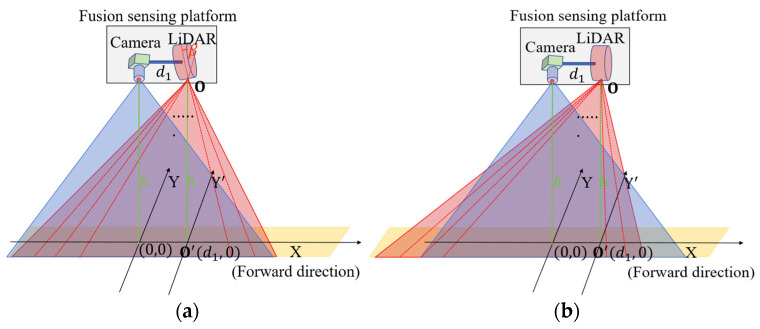
The schematic diagram showing the side view of the LiDAR–camera sensor’s overlap scanning area: (**a**) the LiDAR sensor mounted vertically; (**b**) the LiDAR sensor mounted with an inclination β.

To maximize the LiDAR–camera sensor’s overlap scanning area, the LiDAR sensor should be installed with an inclination angle β due to the deployment distance between the LiDAR sensor and camera, as shown in Figure 5.

Considering the field application in sensor deployment and data acquisition, sensor deployment should comply with the following constraints:

Constraint 1: The minimum length $l_{min}^{c}$ of the camera’s detection range should be greater than the width of the pavement marking $W^{m}$:

(9) $$2h\cdot \tan\left(\frac{\gamma_{min}^{c}}{2}\right)>W^{m}$$

Constraint 2: The sensors’ mounting height h should be higher than the vehicle’s half-tire height $h_{min}$ and lower than the height $h_{max}$ of the vehicle’s mirrors:

(10) $$h_{min}<h<h_{max}$$

Constraint 3: When the LiDAR sensor is mounted with an inclination β, the laser beams of the lowest and the highest channel should be fired on the ground:

(11) $$-90{}^{\circ}<\gamma_{min}^{l}+\beta <\gamma_{max}^{l}+\beta <90{}^{\circ}$$

Constraint 4: The rotation angle α of the LiDAR sensor should be less than 180° to diminish the laser firing to the sky, and the LiDAR sensor’s horizontal scanning range should be greater than the width of the pavement marking $W^{m}$:

(12) 0°<α<180°

(13) $$2h\cdot tan\left(\frac{\alpha }{2}\right)>W^{m}$$

Constraint 5: The $l_{min}^{F}$ value of the detection range after the fusion of the width of the LiDAR–camera overlap area should be greater than the pavement marking width $W^{m}$:

(14) $$\min\left\{y_{max}^{l},y_{max}^{c}\right\}-\max\left\{y_{min}^{l},y_{min}^{c}\right\}>W^{m}$$

Therefore, the final multi-objective optimum model can be expressed as:

(15) $$\left\{\begin{array}{c} \max f_{1}\left(h,\alpha ,\beta \right)=\min\left\{x_{max}^{l},x_{max}^{c}\right\}-\max\left\{x_{min}^{l},x_{min}^{c}\right\}\\ max\;f_{2}\left(h,\alpha ,\beta \right)=\sum_{i=1}^{i=32}\sum_{j=0}^{j=\frac{\alpha }{\theta }}n_{i,j} \end{array}\right.$$

To obtain the unique optimal decision variables h,α,β instead of the Pareto-optimal frontier, objective functions $f_{1}$ and $f_{2}$ are multiplied to obtain the single objective for the optimization mathematical model. Therefore, the LiDAR–camera deployment problem for pavement marking distress fusion detection can be described as

(16) $$\underset{\left(h,\alpha ,\beta \right)}{\max}\begin{array}{c} \left[\left(\min\left\{x_{max}^{l},x_{max}^{c}\right\}-\max\left\{x_{min}^{l},x_{min}^{c}\right\}\right)\times \left(\sum_{i=1}^{i=m}\sum_{j=0}^{j=\frac{\alpha }{\theta }}n_{i,j}\right)\right]\\ s.t.\left\{\begin{array}{c} 2h\cdot \tan\left(\frac{\gamma_{min}^{c}}{2}\right)>W^{m}\\ h_{min}<h<h_{max}\\ -90{}^{\circ}<\gamma_{min}^{l}+\beta <\gamma_{max}^{l}+\beta <90{}^{\circ}\\ 2h\cdot tan\left(\frac{\alpha }{2}\right)>W^{m}\\ 0{}^{\circ}<\alpha <180{}^{\circ}\\ \min\left\{y_{max}^{l},y_{max}^{c}\right\}-\max\left\{y_{min}^{l},y_{min}^{c}\right\}>W^{m} \end{array}\right. \end{array}$$

### 3.2. Solution Algorithm

The onboard LiDAR–camera deployment optimization for pavement marking distress detection is a high-dimensional constrained optimization problem. The RFO algorithm shows its superior capability in balancing global exploration and local exploitation for high-dimensional constrained optimization problems [31,32]. Specifically, the RFO algorithm’s dynamic step-size adjustment mechanism, derived from scent-tracking and ambush behaviors, enables adaptive switching between wide-area search for individual sensors and precision refinement for multi-sensor fusion, providing a non-convex objective landscape for the proposed LiDAR–camera deployment optimization problem [33].

To address the LiDAR–camera deployment optimization problem, multi-dimensional traps were introduced to bound the solution space and guide the search path of the red fox agent. Each decision variable was conceptualized as prey within the optimization landscape, while constraints were modeled as one-dimensional traps. The red fox navigates the constrained search space to capture prey, ensuring alignment with system requirements.

During the prey position update phase, a dynamic mechanism ensures the iterative refinement of solutions. Specifically, if the updated prey position (new candidate solution) yields a superior objective value compared to the current optimal prey, the latter is replaced by the former. This strategy not only maintains solution diversity but also accelerates convergence toward globally optimal configurations. By integrating multi-dimensional constraints into the hunting metaphor, the algorithm effectively balances exploration and exploitation, avoiding local optima while adhering to physical and operational deployment limits. The processes of the proposed improved RFO algorithm are as follows:

Step 1: The red fox global search phase. In the global search phase, each candidate solution $x_{i,t}$ operates independently within the prey population. The population is first ranked in ascending order based on objective function values to identify the optimal candidate solution $x_{best,t}$, which serves as a dynamic reference point for subsequent iterations. For each candidate solution $x_{i,t}$, the Euclidean distance to $x_{best,t}$ is computed as follows:

(17) $$x_{best,t}=\left\|x_{i,t}-x_{best,t}\right\|$$

Then, the D value is set, and scaling parameters are randomly chosen:

(18) $$\alpha_{d}\in \left(0,d_{i,t}\right),\;d=\left[1,2\ldots ,D\right]$$

If $x_{i,t,global}^{D}$ satisfies the constraints, then the red fox individual moves to point $x_{i,t,global}$:

(19) $$x_{i,t,global}=\left[x_{i,t,global}^{1},x_{i,t,global}^{2}\ldots ,x_{i,t,global}^{D}\right]$$

(20) $$x_{i,t,global}^{D}=x_{i,t}^{D}+\alpha_{d}\cdot sign\left(x_{i,t}^{D}-x_{best,t}^{D}\right)$$

If $x_{i,t,global}^{D}$ does not satisfy the constraint, $x_{best,t}$ is updated by setting the sorted second $x_{best-1,t}$ to the new $x_{best,t}$.

Step 2: The red fox localized search phase. The local search phase is initiated immediately after the global search phase and employs a modified snail helix equation to simulate the red fox’s stealthy approach toward its prey. The execution of this phase is governed by a stochastic threshold mechanism: for each candidate solution $x_{i,t}$, a uniformly distributed random value $\mu_{i,t}\in (0,1)$ is generated. If $\mu_{i,t}<0.75$, the local search phase is omitted. This probabilistic bypass mimics scenarios where the red fox is detected by its prey, prompting a strategic pause to avoid premature pursuit and conserve energy. Conversely, if $\mu_{i,t}\geq 0.75$, the algorithm simulates the red fox advancing toward the prey, refining the solution through localized exploitation. This behavior is mathematically expressed as:

(21) $$x_{i,t+1}=x_{i,t}+∆x\cdot \cos\left(\theta \right)$$

where ∆x and θ are the step size and angular displacement, respectively. By adaptively triggering local search based on $\mu_{i,t}$, the algorithm balances exploration and exploitation, ensuring robust convergence while avoiding local optima.

Step 3: The prey location update phase. During the predation phase, the algorithm iteratively refines the candidate solution population to enhance convergence efficiency. The lowest 5% of solutions, ranked by fitness value, are systematically eliminated to remove suboptimal candidates. These positions are then replenished with new candidate solutions generated stochastically, ensuring population diversity and preventing premature stagnation.

Subsequently, a dynamic spherical search subspace is defined to focus exploration on promising regions. The subspace is characterized by its centroid $x_{center,t}$, derived from the current population’s spatial distribution, and its adaptive diameter $d_{area,t}$, which scales with the problem’s dimensionality and convergence progress. Mathematically, the spherical subspace $S_{t}$ is expressed as

(22) $$S_{t}=\left\{x\in R^{n}|\left\|x-x_{center,t}\right\|\leq \frac{d_{area,t}}{2}\right\}$$

(23) $$d_{area,t}=\left\|x_{best1,t}-x_{best2,t}\right\|$$

This adaptive bounding mechanism ensures a balanced trade-off between global exploration and local exploitation, guiding the search toward high-fitness regions while maintaining algorithmic robustness in complex optimization landscapes.

For each newly generated candidate solution $x_{j,t}$, a uniformly distributed random value $k_{j,t}\in \left(0,1\right)$ is independently sampled to govern its placement. This probabilistic threshold mechanism balances exploration and exploitation as follows:

(1) Exploration-Driven Placement ($k_{j,t}>0.45$): The candidate solution $x_{j,t}$ is randomly positioned within the global search space, explicitly excluding the predefined spherical subspace $S_{t}$. This ensures a broad exploration of unexplored regions while avoiding redundancy in localized search areas.

(2) Exploitation-Driven Placement ($k_{j,t}\leq 0.45$): The candidate solution $x_{j,t}$ is derived from a weighted combination of two elite solutions, $x_{best1,t}$ and $x_{best2,t}$, to intensify search efforts near high-fitness regions:

(24) $$x_{j,t}=\frac{k_{j,t}{\cdot x}_{best1,t}+x_{best2,t}}{2}$$

where $k_{j,t}$ introduces stochasticity to diversify exploitation, while the elite solutions guide convergence toward optimal regions. $x_{j,t}$ needs to satisfy the constraints.

The calibrated threshold of 0.45 ensures a bias toward exploration (55% probability) while retaining targeted exploitation (45% probability), fostering a robust balance between global diversification and local intensification.

Upon generating new candidate solutions (prey), their fitness values are evaluated against the current optimal solution. If the fitness of the newly generated prey surpasses that of the incumbent optimal solution (i.e., the new solution exhibits improved performance), the position of the current optimal prey is updated to reflect the superior candidate:

(25) $$x_{best,t}\leftarrow x_{j,t}\;if\;f\left(x_{j,t}\right)<f\left(x_{best,t}\right)$$

where $f\left(\cdot \right)$ is the objective function. This elitist strategy ensures that the algorithm retains the best-known solution at each iteration, progressively refining convergence toward the global optimum while maintaining computational efficiency.

## 4. Case Study

### 4.1. Experimental Setup

**Figure 6:**
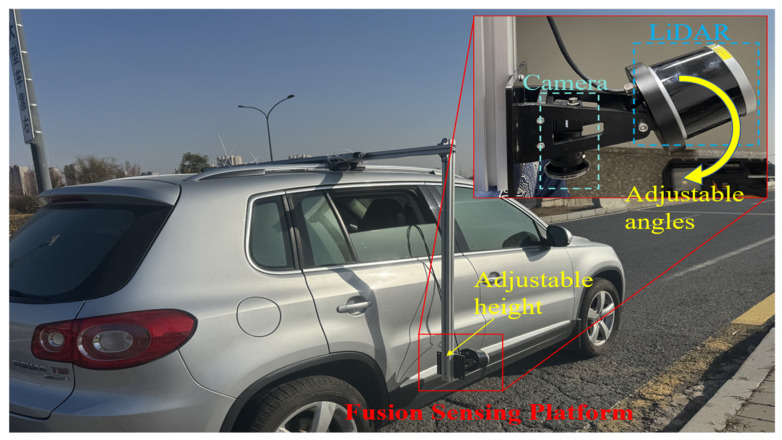
The snapshot of onboard LiDAR–camera sensor deployment.

The RoboSense, Shenzhen, China—RS-Helios-5515 LiDAR sensor and the Hikvision, Hangzhou, China—MV-CU03-A0GM/GC camera were deployed on a Saic Volkswagen, Shanghai, China—Tiguan SUV vehicle to test the effectiveness of the proposed optimized deployment method, as shown in Figure 6.

The built-in and setting parameters of the LiDAR and camera sensors in the experiments are shown in Table 1.

### 4.2. Comparison of Solution Algorithms

To evaluate the performance of the proposed solution algorithm, the RFO algorithm, the Genetic Algorithm (GA) [34], the Particle Swarm Optimization (PSO) [35], and a global traversal algorithm were used to compare the solution results and convergence rates. In the experiments, the GA’s crossover probability and probability of mutation were set as 0.8 and 0.3, respectively, according to Ref. [34]. The cognitive and social weights of PSO were 0.5 and 1 according to Ref. [35].

**Figure 7:**
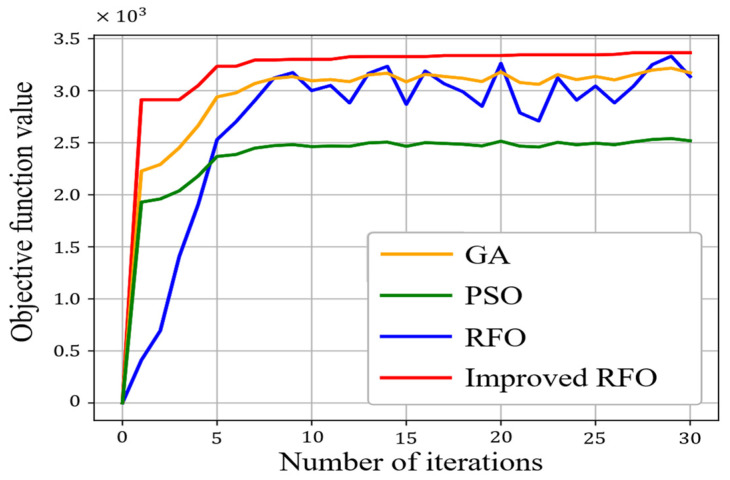
The convergence rates of different algorithms.

The design principles and mechanisms of the RFO algorithm provide it with unique advantages when addressing complex optimization problems. For instance, this algorithm exhibits strong global search capabilities while maintaining good accuracy during local searches. This characteristic enables the RFO algorithm to effectively avoid becoming trapped in local optima when confronted with multimodal and nonlinear optimization problems. By comparing it with the GA and PSO, we aim to illustrate the potential advantages of the RFO algorithm in terms of solution quality and convergence speed, thereby providing strong support for its application in sensor layout optimization. The comparison results are shown in Table 2 and Figure 7.

The improved RFO algorithm, augmented with multi-dimensional trap constraints, exhibits accelerated convergence compared to baseline methods. By the 20th iteration, the algorithm approaches near-optimal solutions, achieving rapid progress toward global optima. Furthermore, the improved RFO guarantees monotonic improvement across iterations: each subsequent prey update is constrained to yield a solution no worse than the current optimal prey. This elitist update mechanism ensures that iterative results strictly dominate prior solutions, a property not inherently upheld by the RFO algorithm, GA, or PSO.

Notably, while the improved RFO algorithm produces optimization results equivalent to exhaustive global traversal (i.e., achieving the highest fitness values), it accomplishes this with a significantly reduced computational time. Although PSO retains a marginal speed advantage, its solutions exhibit markedly inferior fitness values compared to the improved RFO algorithm. This underscores a critical trade-off: the enhanced RFO algorithm prioritizes solution quality over raw computational speed, making it preferable for applications demanding high precision.

### 4.3. Field Test with Optimum Solution

**Figure 8:**
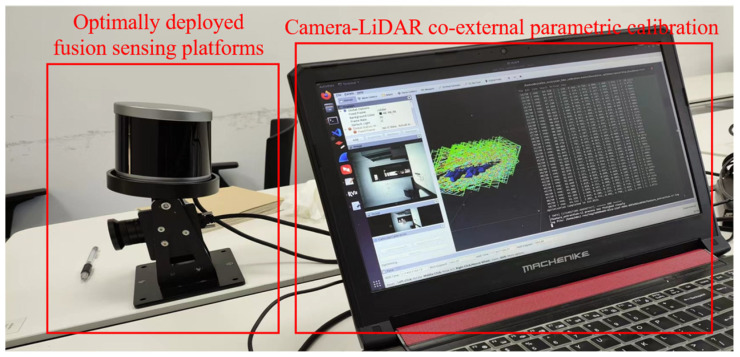
The snapshot of LiDAR–camera spatiotemporal calibration in robot operating system.

Before the onboard LiDAR–camera platform was installed on a vehicle to collect pavement marking data. LiDAR and camera data were spatiotemporally calibrated in a robot operating system (ROS) to achieve data timestamp synchronization and coordinate alignment, leveraging the sensor’s internal and external parameters [36], as shown in Figure 8.

In terms of pavement materials, there are significant differences between asphalt and concrete roads in optical properties and surface roughness. Asphalt roads typically have a higher surface roughness and lower reflectivity, which makes the contrast with pavement markings more pronounced, facilitating the extraction and detection of these markings and thus improving detection accuracy. Conversely, concrete roads tend to be smoother and have higher reflectivity, which can hinder the extraction and detection of line markings.

Secondly, the differences between thermoplastic pavement markings and paint film pavement markings should not be overlooked. Thermoplastic line markings possess strong wear resistance and better reflective properties, allowing them to maintain high visibility under various weather conditions. This enables LiDAR sensors to more clearly identify the markings during detection, thereby reducing the likelihood of misjudgment. In contrast, paint film pavement markings may be inferior to thermoplastic materials regarding durability and reflectivity, particularly under long-term use and high traffic conditions, which could lead to a gradual decline in performance and consequently reduce the reliability of LiDAR sensor detection.

**Figure 9:**
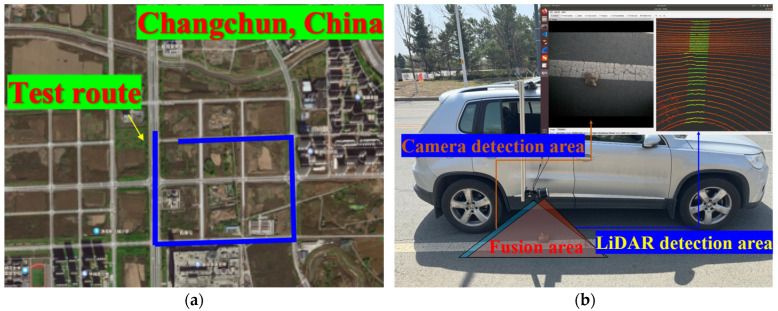
The snapshot of field test experiments: (**a**) the field test route in the experiments; (**b**) the onboard LiDAR–camera deployment based on optimization results.

Experiments were conducted to verify the effectiveness of the proposed LiDAR–camera deployment optimization method for pavement marking distress fusion detection. The field test experiments, conducted on Eco Street in Changchun city, China, which features asphalt pavement and thermoplastic material, took place under conditions of partial shadowing, no precipitation, and with road markings that were mostly intact, although some were damaged as shown in Figure 9.

**Figure 10:**
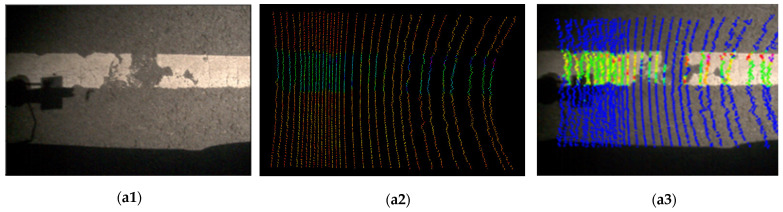

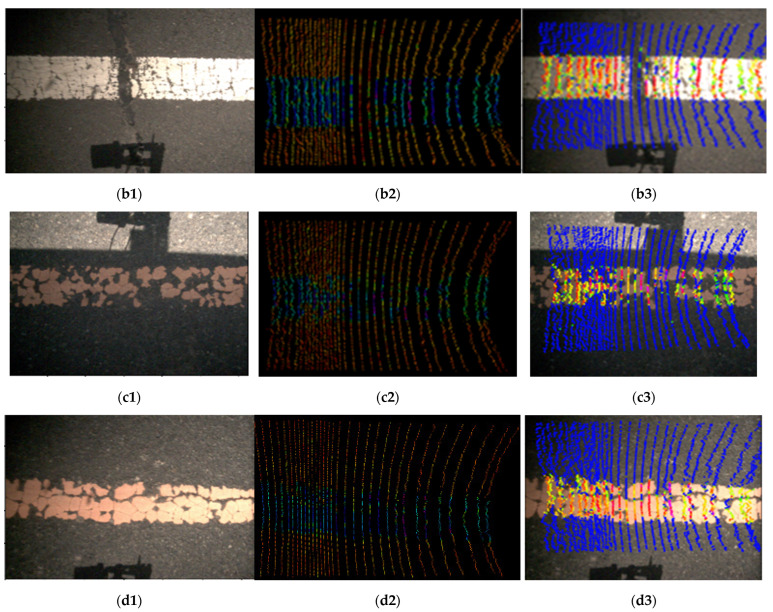
The pavement marking detection results under different shadow and light conditions: (**a1**–**d1**) images extracted from the camera; (**a2**–**d2**) the point clouds extracted from the LiDAR sensor; (**a3**–**d3**) LiDAR–camera data fusion for pavement marking distress detection.

To evaluate the reliability of the proposed LiDAR–camera optimization deployment method for pavement marking distress fusion detection, the pavement marking detection length $f_{1}$ and the point cloud falling on the pavement marking $f_{2}$ of the ground truth in field tests and the proposed mathematical model were compared. The results are shown in Table 3 and Figure 10.

In the field test, experiments were conducted to collect data on the white and yellow pavement markings under shadows and different lighting conditions. This phenomenon degrades the accuracy of image-based detection methods due to reduced contrast and false feature extraction. However, integrating LiDAR point clouds mitigates shadow-induced interference by supplementing 3D spatial data, which remains invariant to lighting variations.

In addition, LiDAR–camera data fusion can capture richer marking data while demonstrating strong anti-interference capability. This synergistic approach ensures the reliable detection of pavement marking distress and provides a robust foundation for subsequent fusion pavement marking distress detection algorithms under challenging environments.

Furthermore, the results of the field test and mathematical model comparison reveal a relative error of less than 7%, demonstrating a high degree of concordance. This narrow margin of error empirically validates the effectiveness of the proposed method in real-world scenarios, confirming its practical applicability for pavement marking distress fusion detection.

## 5. Conclusions

In this study, an onboard LiDAR–camera deployment optimization method was proposed to address the shadows and light changes in pavement marking distress detection by taking the strengths of individual sensors. First, the LiDAR and camera sensor’s scanning range was assessed according to its inherent characteristics. Then, LiDAR–camera deployment optimization was mathematically formulated to achieve the largest overlap scanning area and point cloud density on pavement markings for distress fusion detection. Finally, an improved RFO algorithm was developed to speed up the convergence rate and optimize the solution result by leveraging a multi-dimensional trap mechanism and an improved prey position update strategy.

The experimental results show that the proposed solution method achieves a shorter time of 22.50 s in solving optimization deployment parameters with a mounting height, LiDAR rotation angle, and LiDAR installation inclination of 0.53m, 60°, and 12°, respectively. With the optimization deployment solution, the field test results show that the relative error between the mathematical model evaluation and field test measurements is less than 7%, achieving 5127 LiDAR points falling on a 0.58 m pavement marking per data frame for fusion distress detection. The experiments also demonstrated the reliability and robustness of LiDAR–camera fusion detection in real-world scenarios, under different shadow and light conditions.

However, this study is not free from limitations. (1) The proposed method was tested on straight pavement markings. However, there are curved roads and roundabouts in the real world. Therefore, the proposed method should be further tested on curved pavement markings. (2) In the experiments, the LiDAR sensor RS-Helios-5515 and the camera sensor MV-CU03-A0GM/GC were used to test and evaluate their performance. In future studies, more types and brands of LiDAR and camera sensors should be tested to determine a cost-effective sensor for pavement marking distress fusion detection. (3) LiDAR–camera fusion data for pavement marking detection in different shadow and light conditions was shown in this study. More pavement marking distress fusion detection and evaluation algorithms will be developed in future work. (4) Another limitation is that this study was conducted using a specific experimental vehicle. In future studies, we will consider conducting testing on other types of experimental vehicles to change the constraints of the mounting brackets and installation heights, enabling broader detection capabilities.

## Figures and Tables

**Table 1:** The built-in and setting parameters of the LiDAR and camera sensors in the experiments.

Sensors	Features	Parameters
Camera	Frame Rate	201.4 FPS
	Resolution	1280 × 1024
	Focal Length	3.5 mm
	Angle of View	H: 82.6°; V: 70.2°
LiDAR	Horizontal FOV	360°
	Vertical FOV	−55° ~ +15°
	Frame Rate	10 Hz ~ 20 Hz
	Channels	32
	Vertical Resolution	1.44° ~ 2°
	Horizontal Resolution	0.2° ~ 0.4°

**Table 2:** The LiDAR and camera sensor’s deployment parameters solved using different algorithms.

Algorithms	Sensor Deployment Parameters			Fitness	Running Time (s)
	h (cm)	α (°)	β (°)		
Global traversal	0.53	60	12	3406.9	1127.38
GA	0.55	60	12	3207.2	105.38
PSO	0.59	60	6	2502.8	18.06
RFO	0.54	60	10	3195.4	37.1
Improved RFO	0.53	60	12	3406.9	22.50

**Table 3:** The comparison of the optimization objective between the field tests and mathematical model.

Objectives	Field Test	Mathematical Model	Error (%)
Length (m)	0.58	0.62	6.45
Point Number	5127	5495	6.70

## Data Availability

The data used to support the findings of this study are available from the corresponding author upon request.
